# Inflammatory Cells in Control and Prolapsed Uterosacral Ligament Tissue

**DOI:** 10.1007/s43032-024-01618-4

**Published:** 2024-06-21

**Authors:** David J. Orlicky, E. Erin Smith, Joshua Johnson, Ashley E. Hilton, Marsha K. Guess, Lauren G. Rascoff, Jaime S. Arruda, Juana A. Hutchinson-Colas, Ivana Yang, Kathleen A. Connell

**Affiliations:** 1 Department of Pathology, University of Colorado School of Medicine, Aurora, CO USA; 2 Department of Obstetrics and Gynecology, University of Colorado School of Medicine, Aurora, CO USA; 3 Department of Obstetrics, Gynecology and Reproductive Sciences, Robert Wood Johnson Medical School, Rutgers Health, New Brunswick, NJ USA; 4 Department of Biomedical Informatics, University of Colorado School of Medicine, Aurora, CO USA

**Keywords:** Pelvic organ prolapse, Uterosacral ligament, Neutrophils, Lymphocytes, Macrophages, Mast cells

## Abstract

Pelvic organ prolapse (POP), a downward descent of the vagina and/or uterus through the vaginal canal, is a prevalent condition affecting up to 40% of women. Several risk factors of POP have been identified, including childbirth, connective tissue defects, and chronic intra-abdominal pressure; however, the underlying etiologies of POP development are not fully understood, leading to a high burden on patients and the healthcare systems. The uterosacral ligaments are key support structures of the uterus and upper vagina. Our previous work describes observed histopathological changes in uterosacral ligament (USL) tissue and demonstrates the presence of neutrophils in a subgroup of POP individuals. This presence of neutrophils prompted an examination for the presence of a broader spectrum of inflammatory cell types in the USL. Immunohistochemical staining was performed to identify neutrophils, lymphocytes, macrophages, and mast cells outside of the vasculature. All 4 inflammatory cell types were increased in the POP-HQ system-defined POP-Inflammatory (POP-I) phenotype USL tissue relative to the USL tissues of control or other POP-HQ phenotypes. Focal T-lymphocyte and macrophage co-accumulations were observed in the arterial walls from some patients of the POP-vascular (POP-V) phenotype suggesting previous arterial injury. In addition, 1 control and 2 POP-V subjects’ USLs contained arterial wall foamy macrophages, evidence of atherosclerosis. These findings further support a complex etiology for POP and indicate that personalized approaches to preventing and treating the condition may be warranted.

**Supplementary Information:**

The online version contains supplementary material available at 10.1007/s43032-024-01618-4.

## Introduction

Pelvic organ prolapse (POP) is one of the complex, multifactorial pelvic floor disorders (PFD),where loss of pelvic support results in protrusion of the vagina, and pelvic organs. Despite the high prevalence of POP, our understanding of the mechanism(s) underpinning POP is limited, leading to exceedingly high costs on the healthcare system and significant physical and emotional burden on affected women’s quality of life. Nearly one quarter of adult women in the United States will experience at least one PFD in their lifetime, with numbers anticipated to increase substantially over the next 30 years given the changing demographics and aging of the US population. [1, 2]. Clinically, pelvic prolapse is characterized by progressive loss of pelvic organ support leading to descent of those organs into the vagina and beyond [3]. Advanced prolapse is associated with structural deficiencies of the uterosacral-cardinal ligament complex, including the uterosacral ligament (USL) which helps to provide apical support to the vagina [4, 5]. The main known risk-factors for POP include aging, parity with vaginal delivery, increased body-mass-index (BMI), and menopause [6, 7].

Multiple associations suggest the involvement of inflammation in the etiology POP. For instance, the incidence of POP is higher in human patients with the autoinflammatory disease ankylosing spondylitis, a condition involving activated neutrophils, macrophages, and possibly B-lymphocytes [8]. Transcriptomic studies using tissue from human POP patients have identified an upregulated immune response [9–11]. In studies designed to examine possible similarities between human POP patients and the Loxl1 gene-deficient mouse prolapse model, prolapsed human pelvic floor tissue transcriptomic data revealed a high level of T-cell inflammatory response genes while Loxl1-KO mouse pelvic tissue showed an acute inflammatory response with T cell signatures [12]. Lastly, a microarray dataset from human POP patients found the signature of an infiltration of activated mast cells and neutrophils [13]. However, not all previous studies have identified an inflammatory component to POP [14, 15].

Preventative and treatment strategies of POP are limited due to a lack of understanding of its multiple etiologies. In our previous work, we developed the Pelvic Organ Prolapse – Histologic Quantification (POP-HQ) system [16] to systematically evaluate the histologic components of USLs to better understand the various changes in cellular and extracellular content in women with POP to better understand the etiologies of POP. We demonstrated that semi-quantitative histopathological scoring followed by principal component analysis can separate the USLs from women with POP into 3 distinct phenotypes that may correspond to different etiologies. These USL histopathological phenotypes include one with an increase in neutrophils (POP-I), a second with increased collagen and adipose deposition (POP-A), and a third with neointimal hyperplasia (NIH) vascular changes (POP-V).

Based upon our results in the POP-HQ system, we pursued a more comprehensive investigation of the presence of inflammatory cell types and their locale within USL tissue. We have used an immunohistochemical approach to directly identify and allow the quantification of and localization of the presence of several inflammatory cell types. These investigations revealed an increase in the levels of multiple types of inflammatory cells in the USL tissue of patients with the POP-I phenotype but not in other phenotypes of POP. Our studies further support the segregation of POP USL-tissue into specific phenotypes and represents an additional step towards understanding POP etiology, and potentially, towards the personalization of POP treatment [17].

## Materials and Methods

A Strengthening The Reporting of OBservational Studies in Epidemiology (STROBE) checklist was used to guide the experimental design and reporting of the demographic data presented here [18].

### Specimen Collection

USL biopsies were obtained as previously published from women undergoing hysterectomy for POP or for benign gynecological conditions (i.e., abnormal uterine bleeding, symptomatic fibroids) [16] with approval of the Colorado Multiple Institution Review Board (COMIRB #15–2245) and the Rutgers Institutional Review Board (#Pro20160001312). Written informed consent was obtained from all patients. Exclusion criteria included current pregnancy, previous POP or urinary incontinence surgery, or malignancy. Menopause was defined as no menses within the past year or previous oophorectomy. Women underwent pelvic exam and were quantified as POP or control using the Pelvic Organ Prolapse Quantification System (POP-Q) [19]. Women with stage II or greater descent were assigned to the POP group and women with stages 0 and I POP were assigned to the control group. Demographic data (Table 1) were collected from each subject and have been uploaded to Figshare: https://figshare.com/s/365b699d5d8078c90ff0.

**Table 1:** Summary of the Major Demographics for the Control and POP-phenotypes subjects. Average and standard deviations of the mean are included for the main demographic features for each phenotype of subjects. The N of each group is indicated. Statistical comparisons of the inflammatory cell IHC data for all phenotypes of subjects were performed using R software with required packages indicated where appropriate [20]. Welch’s unequal variances t-test was used when the phenotypes had unequal variances and were of unequal sample size. Analysis of variance with Tukey’s post-hoc pairwise test was used when making multiple comparisons between continuous variables and Pearson's Chi-squared test with Yates' continuity correction for categorical variables (R package ‘RVAideMemoire’). Mean values that significantly differ from one another by group are denoted by letters (a, b, c), *P* ≤ 0.05

	Control	POP-A(DIPOSE)	POP-I(NFLAM)	POP-V(ASCULAR)
*N*	21	19	22	16
Feature	Mean ± SD	Mean ± SD	Mean ± SD	Mean ± SD
Age	45.7 ± 9.3 ^a^	58.6 ± 12.1 ^b^	57.1 ± 13.5 ^b^	63.4 ± 11.2 ^b^
Race and Ethnicity	81% White; 5% Native Hawaiian or other Pacific Islander; 14% not indicated	95% White; 5% Native Hawaiian or other Pacific Islander	95% White; 5% not indicated	44% White; 31% Hispanic or Latino; 19% Asian; 6% Black or African-American
Vaginal Deliveries	1.8 ± 1.6 ^a^	2.8 ± 1.0 ^b^	2.4 ± 0.9 ^a^	3.0 ± 1.4 ^b^
BMI	27.8 ± 5.9 ^a^	26.3 ± 5.8 ^a^	27.4 ± 4.6 ^a^	29.0 ± 6.0 ^a^
Post-Menopausal	4 (19%) ^a^	14 (74%) ^b^	14 (64%) ^b^	12 (75%) ^b^
Smoking status, *n* (%)	7 yes (33%) ^a^	5 yes (26%) ^a^	12 yes (55%) ^a^	4 yes (25%) ^a^
Anti-Inflam meds; none	8 (38.1%) ^a^	8 (42.1%) ^a^	10 (45.4%) ^a^	8 (50.0%) ^a^
Anti-Inflam; ASA, motrin	7 (33.3%) ^a^	5 (26.3%) ^a^	5 (20.8%) ^a^	5 (31.3%) ^a^
Anti-Inflam; levothyroxine, hydroxychloroquine	2 (9.5%) ^a^	4 (23.5%) ^a^	7 (29.2%) ^a^	4 (25.0%) ^a^
Taking non-inflammatory related medicines	5 (23.8%) ^a^	5 (26.3%) ^a^	8 (36.4%) ^a^	3 (18.8%) ^a^

During surgery, a 0.5 cm segment of each USL was biopsied within 1 cm of their cervical insertion and placed in neutral buffered formalin, paraffin embedded, sectioned, and trichrome stained. The specimens were then reviewed by a trained histopathologist (DJO) in a blinded fashion. Utilizing the semi-quantitative histopathological POP-HQ scoring system [16] to assess each USL, the full extent of each USL-section was examined and scored. All POP-HQ data (Table 2) for each subject have been uploaded to Figshare: https://figshare.com/s/365b699d5d8078c90ff0.

**Table 2:** Summary of the POP-HQ histopathological scoring for the Control and POP-phenotype Subgroups. Average and standard deviations of the mean are included for the POP-HQ histopathological scoring features for each phenotype of subjects. The N of each group is indicated. Statistical comparisons of POP-HQ phenotypes’ histopathological scoring data were performed using R software with required packages indicated where appropriate [20]. Welch’s unequal variances t-test was used when the phenotypes had unequal variances and were of unequal sample size. Analysis of variance with Tukey’s post-hoc pairwise test was used when making multiple comparisons between continuous variables and Pearson's Chi-squared test with Yates' continuity correction for categorical variables (R package ‘RVAideMemoire’). Mean values that significantly differ from one another by group are denoted by letters (a, b, c), *P* ≤ 0.05

	Control	POP-A(DIPOSE)	POP-I(NFLAMMATORY)	POP-V(ASCULAR)
*N*	21	19	22	16
Feature	Mean ± SD	Mean ± SD	Mean ± SD	Mean ± SD
Fibrillar Collagen	53.3 ± 17.0 ^a^	62.8 ± 18.5 ^a^	53.5 ± 17.4 ^a^	49.5 ± 23.5 ^a^
Total Non-Vascular Smooth Muscle	2.3 ± 0.7 ^a^	1.3 ± 0.7 ^b^	1.9 ± 0.7 ^a^	2.1 ± 0.5 ^a^
SmFDO	0.7 ± 0.4 ^a^	1.3 ± 0.5 ^b^	1.3 ± 0.5 ^b^	0.8 ± 0.5 ^a^
Muscle Fiber Vesicles	0.1 ± 0.1 ^a^	0.1 ± 0.2 ^a^	0.0 ± 0.1 ^a^	0.2 ± 0.4 ^a^
Adipose	0.5 ± 0.8 ^a^	1.0 ± 0.8 ^a^	0.5 ± 0.6 ^a^	0.2 ± 0.3 ^b^
PMN-Inflam	0.5 ± 0.4 ^a^	0.4 ± 0.3 ^a^	2.0 ± 0.6 ^b^	0.0 ± 0.1 ^c^
PN-Inflam	0.1 ± 0.3 ^a^	0.1 ± 0.1 ^a^	1.0 ± 0.9 ^b^	0.0 ± 0.0 ^a^
NIH	0.4 ± 0.7 ^a^	0.1 ± 0.3 ^a^	0.0 ± 0.1 ^a^	2.3 ± 0.7 ^b^
Vessel Quantity	1.1 ± 0.8 ^a^	0.7 ± 0.6 ^a^	1.0 ± 0.5 ^a^	2.0 ± 0.5 ^b^

### Immunohistochemistry

A list of the antibodies used, the manufacturer, the antibody titer, and specifics about the immunohistochemistry methods are listed in Table 3. The histological quantification criteria used for the IHC staining of neutrophils, T cells, B cells, mast cells, dendritic cells, and macrophages are indicated in Table 4. Importantly, only inflammatory cells that were outside of vessels were counted. All immunohistochemistry results data for each subject has been uploaded to Figshare: https://figshare.com/s/365b699d5d8078c90ff0

**Table 3:** The antibodies and staining methods used in this study

Antibody	Company	Catalog #	dilution	Qty/sld	Antibody Incub	Retrieval method	Stainer	Detection
Anti-myelo peroxidase	Abcam	ab 208,670	1:1500	100 ul	32’, 37 C	BORG 10′ 110 C in NexGen Pressure cooker (Biocare)	Roche (Ventana) Discovery Ultra	ChromoMap Inhibitor 12', OmniMAP polymer Rb-HRP 12', ChromoMap DAB kit 12'
Anti-CD3	DAKO	A452	1:500	200 ul	60’, R.T	Citrate 10′ 110 C in NexGen Pressure cooker (Biocare)	Benchtop—Rabbit ImmPress (Vector)	10' H2O2, 20' NHS, 60' ab, 30' Rb ImmPress, 5' DAB
Anti-CD68	DAKO	M0814	1:750	100 ul	32’, 37 C	Citrate 10′ 110 C in NexGen Pressure cooker (Biocare)	Roche (Ventana) Discovery Ultra	UltraView DAB kit
Anti-CD20cy	DAKO	M0755	1:300	100 ul	32’, 37 C	Citrate 10′ 110 C in NexGen Pressure cooker (Biocare)	Roche (Ventana) Discovery Ultra	UltraView DAB kit
Anti-PD1	Spring Bioscience	M5690	1:200	200 ul	32’, 37 C	BORG 10′ 110 C in NexGen Pressure cooker (Biocare)	Roche (Ventana) Discovery Ultra	UltraView DAB kit
Anti-CD206	My Biosource	MBS 249,782	1:1000	100 ul	16’, 37 C	Citrate 10′ 110 C in NexGen Pressure cooker (Biocare)	Roche (Ventana) Discovery Ultra	UltraView DAB kit
Anti-CD11c	Cell Signaling	45,581	1:200	100 ul	32’, 37 C	Citrate 10′ 110 C in NexGen Pressure cooker (Biocare)	Roche (Ventana) Discovery Ultra	UltraView DAB kit
Anti-CD56	Cell Marque	MRQ-42	1:1600	100 ul	16’, 37C	UltraCC2 for 8’, 91 C	Roche (Ventana) Discovery Ultra	UltraView DAB kit

**Table 4:** The Histologic Quantification criteria used for each cell type in this study

Antibody	Cell type	Score = 0	Score = 1	Score = 2	Median number of fields counted
Anti-myelo-peroxidase	Neutrophils	< 8 cells/200 × field	8–25 cells/200 × field	> 25 cells/200 × field	15
Anti-CD3	T-lymphocytes	< 5 cells/200 × field	5–15 cells/200 × field	> 15 cells/200 × field	15
Anti-CD68	macrophages	< 3 cells/200 × field	3–10 cells/200 × field	> 10 cells/200 × field	15
Anti-CD20cy	B-lymphocytes	< 3 cells/200 × field	3–10 cells/200 × field	> 10 cells/200 × field	15
Anti-PD1	Activated lymphocytes	< 5 cells/200 × field	5–15 cells/200 × field	> 15 cells/200 × field	15
Anti-CD206	Resolving macrophages	< 3 cells/200 × field	3–10 cells/200 × field	> 10 cells/200 × field	15
Anti-CD11c	Dendritic cells	none/200 × field	≥ 1 cell/200 × field		15
Anti-CD56	NKT cells	Count positive cells / 200 × field			15
Toluidine Blue staining	Mast cells	< 2 cells/400 × field	2–6 cells/400 × field	> 6 cells/400 × field	35

### Data Analysis

Composition and inflammatory cell data were compared between the different POP phenotypes and control. Inflammatory cell data was examined by principal component analysis using R packages ‘factoextra’, ‘fviz_pca’, ‘prcomp’, and ‘princomp’ to compare the extent of the inflammatory cell presence within the POP phenotypes [20]. R software was used for all statistical analyses, with required packages indicated where appropriate (R package) [20]. Welch’s unequal variances t-test was used when the phenotypes had unequal variances and were of unequal sample size. Analysis of variance with Tukey’s post-hoc pairwise test was used when making multiple comparisons between continuous variables. Pearson's Chi-squared test with Yates' continuity correction for categorical variables was used where required (R package ‘RVAideMemoire’). For statistical comparisons of data, *p* < 0.05 was considered significant.

## Results

The cohort for our inflammatory cell assessment included 21 control and 57 prolapsed individuals that were separated by the POP-HQ system into 19 POP-adipose (POP-A), 22 POP-inflammation (POP-I), and 16 POP-vascular (POP-V). Subjects were chosen for the current study, based upon the integrity of the USL tissue, the completeness of the subject’s demographic data, and their POP-HQ phenotype.

### Subject Demographics

Table 1 shows the patient demographics means and intergroup statistics, according to POP-HQ phenotypes, of the subjects used for investigation of inflammatory cell types. Subjects in the POP-A and POP-V phenotypes were older, had more vaginal deliveries, and were more likely to be post-menopausal than control subjects. Subjects in the POP-I phenotype were older and more likely to be post-menopausal than control subjects. Almost all the Control, POP-A and POP-I group subjects identified racially as white, whereas only 44% of the POP-V group did so, the remaining POP-V subjects identified as Hispanic or Latino, Asian, or African American. Importantly, no statistically significant differences were noted in anti-inflammatory drug use between the 4 groups. The demographic data for each subject has been uploaded to Figshare: https://figshare.com/s/365b699d5d8078c90ff0.

### Subjects’ POP-HQ Scoring

Table 2 shows the means, standard deviations, and intergroup statistics for the POP-HQ histopathological scoring features of the subjects used for investigation of inflammatory cell types. The POP-A phenotype had less smooth muscle and more muscle fiber dropout than the control group, the POP-I phenotype had more muscle fiber dropout and inflammation and less NIH than the control group, and the POP-V phenotype had more NIH and less inflammation than the control group. These differences are similar to those we have previously reported for these POP-phenotypes [16]. The POP-HQ data for each subject has been uploaded to Figshare: https://figshare.com/s/365b699d5d8078c90ff0.

### Inflammatory Cell Detection and Quantification

**Fig. 1:**
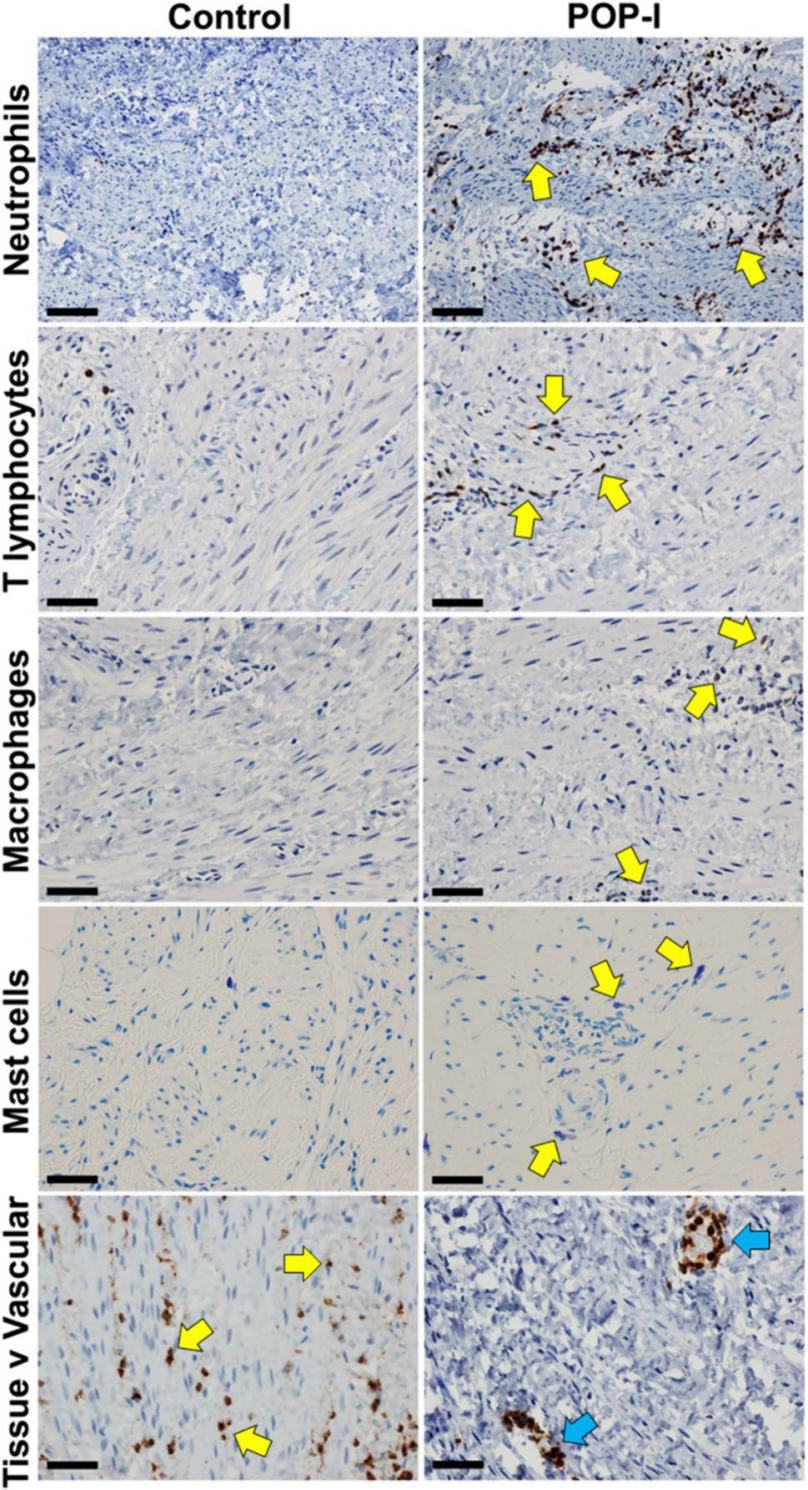
Immunohistochemical staining for inflammatory cells. IHC staining for neutrophils, T-lymphocytes, macrophages, and resolving macrophages are demonstrated, as well as toluidine blue staining of mast cells. B-lymphocytes are not demonstrated due to their very low abundance. All images shown were shot at the same magnification (400x); yellow arrows point to positive cells; size bars are 50 microns.  Data for quantification of all inflammatory cell types are found in Table 5

IHC staining for myeloperoxidase identified neutrophils (Fig. 1, and SupFig 1). Neutrophils were observed as individual cells and clumps and were observed throughout the various USL connective tissues although less commonly inside smooth muscle fascicles and never inside neural bundles. Only neutrophils (and all inflammatory cells) found outside of vessels (Fig. 1, bottom row, left hand side) were considered during quantification scoring (Table 5). The POP-I phenotype had an approximately ten-fold increase in neutrophils compared to the other phenotypes (*p* ≤ 0.05, Table 5). Data for neutrophil quantification for all subjects, as well as the quantification of all other inflammatory cells has been uploaded to Figshare: https://figshare.com/s/365b699d5d8078c90ff0.

IHC staining for CD3 identified T-lymphocytes (Fig. 1, and SupFig 1). Besides their presence in loose connective tissue, T-lymphocytes were observed in all three layers of arterial walls of subjects with NIH ( discussed below). The POP-I phenotype had an approximate doubling of the number of T-lymphocytes than the other phenotypes (*p* ≤ 0.05, Table 5).

IHC staining for CD68 identified all macrophages (Fig. 1, and SupFig 1). Besides their presence in loose connective tissue, macrophages were also observed in the arterial walls of subjects with NIH (Fig. 4, discussed below). The POP-I phenotype had approximately 2–threefold more macrophages than the other phenotypes (*p* ≤ 0.05, Table 5).

Toluidine blue staining was used to identify mast cells (Fig. 1, and SupFig 1). We hypothesized that since mast cells are increased in remodeling tissues, they might be increased in some of the POP-phenotypes. Mast cells were often observed at the interface of different connective tissue types, for instance between adipose tissue and vessels. Subjects in the POP-I phenotype had approximately 50% more mast cells than control subjects but did not have greater numbers of mast cells than POP-A and POP-V subjects (*p* ≤ 0.05, Table 5).

**Table 5:** Summary of the Inflammatory Cell types found in the POP and control USL phenotypes Average and standard deviations of the mean are included for the results of each inflammatory cell IHC for each phenotype of subjects. The N of each group is indicated. Statistical comparisons of the inflammatory cell IHC data for all phenotypes of subjects were performed using R software with required packages indicated where appropriate [20]. Welch’s unequal variances t-test was used when the phenotypes had unequal variances and were of unequal sample size. Analysis of variance with Tukey’s post-hoc pairwise test was used when making multiple comparisons between continuous variables and Pearson's Chi-squared test with Yates' continuity correction for categorical variables (R package ‘RVAideMemoire’). Mean values that significantly differ from one another by group are denoted by letters (a, b, c), *P* ≤ 0.05

		Control	POP-A(DIPOSE)	POP-I(NFLAMMATORY)	POP-V(ASCULAR)
	*N*	21	19	22	16
Cell Type	Cell Marker	Mean ± SD	Mean ± SD	Mean ± SD	Mean ± SD
Neutrophils	Myeloperoxidase	0.1 ± 0.3 ^a^	0.2 ± 0.3 ^a^	1.6 ± 0.4 ^b^	0.0 ± 0.1 ^a^
T-Lymphocytes	CD3	0.3 ± 0.3 ^a^	0.2 ± 0.2 ^a^	0.7 ± 0.3 ^b^	0.3 ± 0.2 ^a^
Macrophages	CD68	0.1 ± 0.1 ^a^	0.1 ± 0.2 ^a^	0.3 ± 0.3 ^b^	0.1 ± 0.1 ^a^
Mast Cells	Toluidine Blue	0.4 ± 0.2 ^a^	0.5 ± 0.2 ^a^	0.6 ± 0.3 ^b^	0.5 ± 0.3 ^a^
B-Lymphocytes	CD20	0.0 ± 0.0 ^a^	0.0 ± 0.1 ^a^	0.0 ± 0.0 ^a^	0.0 ± 0.0 ^a^
Resolving macrophages	CD206	0.1 ± 0.2 ^a^	0.1 ± 0.1 ^a^	0.4 ± 0.3 ^b^	0.2 ± 0.2 ^a^
Dendritic Cells	CD11c	0.0 ± 0.0 ^a^	0.0 ± 0.0 ^a^	0.0 ± 0.1 ^a^	0.0 ± 0.1 ^ a^
NKT Cells	CD56	0.02 ± 0.03 ^a^	0.01 ± 0.02 ^a^	0.04 ± 0.07 ^b^	0.00 ± 0.00 ^a^

IHC staining for CD20 identified B-lymphocytes (SupFig 1). Very few B-lymphocytes were observed in the USL and therefore images of B-lymphocytes are not shown (Fig. 1). No statistical differences were observed in NKT cell numbers between the control and POP-phenotypes (*p* ≤ 0.05, Table 5).

**Fig. 2:**
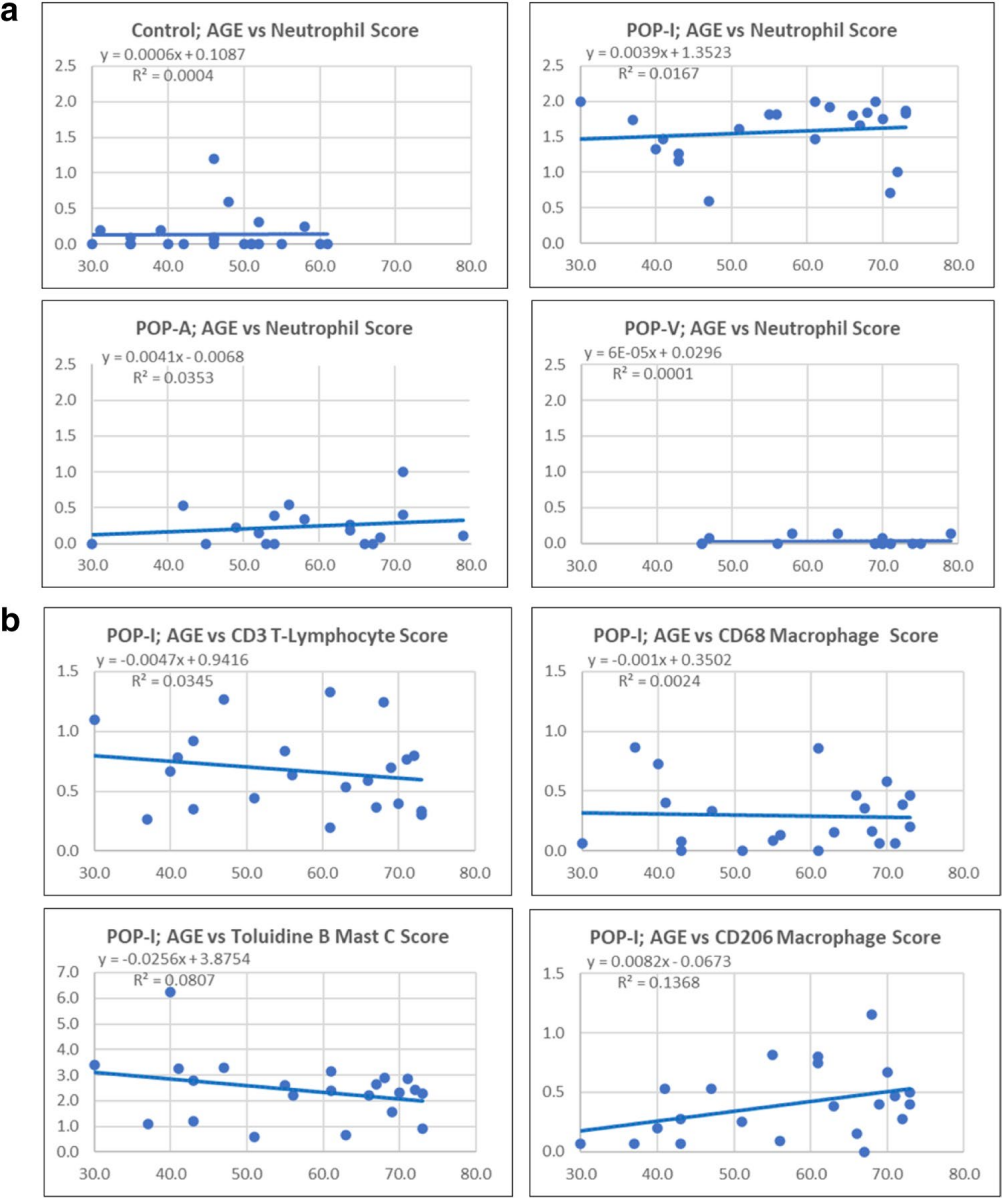
**a** and **b**. Inflammatory Cell Scoring data plotted as a function of Age. **a** Neutrophil scoring data plotted versus age in the various subgroups including control subjects, POP-I subjects, POP-A subjects, and POP-V subjects. Only a very slight increase with age is observed for the POP-I and POP-A subjects. **b** Inflammatory cell scoring data for CD3, CD68, Toluidine blue, and CD206 is plotted vs age for the subgroup POP-I. CD3, CD68, and toluidine blue data do not increase with increasing age whereas the data for CD206 does increase

The hypothesis that inflammageing played a role in the increased levels of inflammatory cells in the POP-I phenotype was examined. In the POP-A and POP-I phenotypes, the relative incidence of neutrophils increased with age although only very slightly between 30 and 70 years of age (Fig. 2a) whereas it did not increase in the control and POP-V phenotypes. The relative incidence of the POP-I phenotype T-lymphocytes, CD68-positive macrophages, and mast cells did not increase with age (Fig. 2b). The relative incidence of CD206-resolving macrophages increased with age but with considerable variation in cell quantity (Fig. 2b).

### Principal Component Analysis

**Fig. 3:**
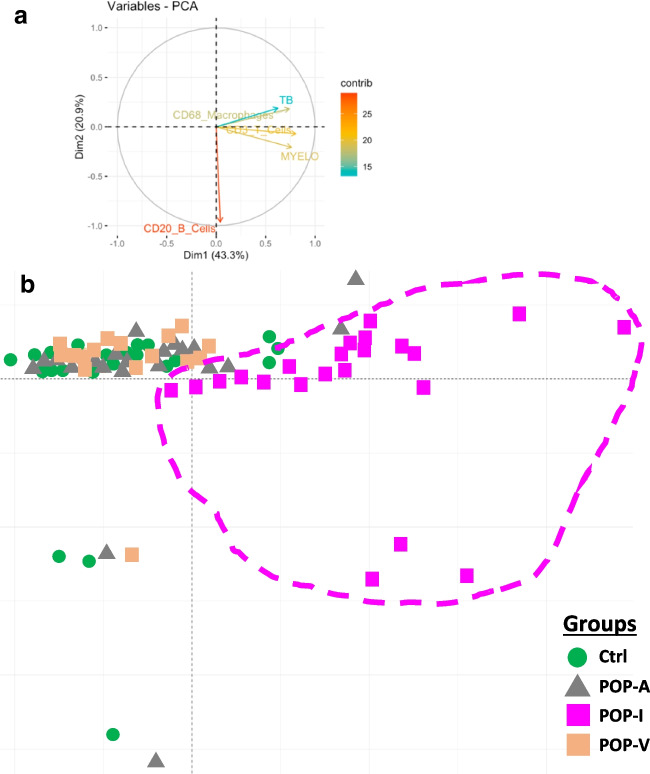
PCA diagram of inflammatory cell abundance data. Shown here is the dimension 1 versus dimension 2 distribution from the PCA using myeloperoxidase, CD3, CD68, toluidine blue, and CD20 quantification data for all the subjects. The vector diagram at the top indicates the relative strength and direction vectors for the data. The dashed outline surrounds the POP-I phenotype subjects (magenta squares) and is included to show how they relatively separate from subjects of the other phenotypes. Control subjects (green circles) completely overlap the POP-A (grey triangles) and POP-V (tan squares) subjects

Results from all patients’ myeloperoxidase, CD3, CD68, CD20, and toluidine blue quantification data were used in principal component analysis (PCA) to determine relatedness or not of USL inflammation. PCA separated all subjects of the POP-I subgroup away from all subjects of the control, POP-A and POP-V phenotypes (Fig. 3) although heterogeneity was observed within the POP-I subgroup.

### Special Stains and Other Findings

**Fig. 4:**
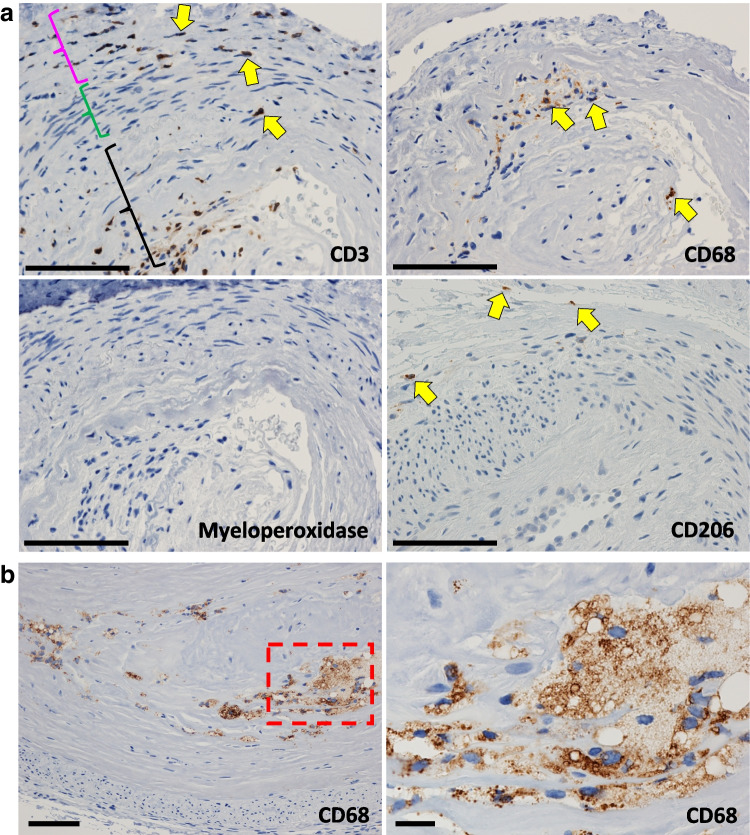
Immunohistochemical staining CD3, CD68 and myeloperoxidase positive cells in the walls of POP-V arteries. **a** IHC staining for CD3 (T-lymphocytes), CD68 (macrophages), and CD206 (resolving macrophages) show their presence in the walls of POP-V arteries and the lack of neutrophils. Abundant tunica intima hyperplasia is also present in these images. The purple bracket denotes the tunica adventitia, green bracket denotes the tunica media, and black bracket denotes the tunica intima. All images shown were shot at the same magnification (400x); Size bars are 200 microns. **b** IHC staining for CD68 (macrophages) shows the presence of “foamy macrophages” in the tunica intima of a few arteries. Right side image is an enlargement of the red boxed area seen in the left-hand image. Size bars: left side, 100 microns, right side, 25 microns

In addition to the quantification of the inflammatory cells, other characteristics of them were of interest. T-lymphocytes and macrophages were observed loosely clustered together in the arterial walls in 5 of the 16 POP-V subjects (31%) showing NIH (Fig. 4a). These co-occurring clusters were also present in 1 of the 21 control subjects. (5%), 0 of the 19 POP-A  subjects (0%), and 0 of the 22 POP-I subjects (0%) (Table 6). CD68 positive cells without the co-occurrence of CD-3 cells were also found loosely associated with the arterial walls in 0 of the 21 Control subjects (5%), 0 of the 19 POP-A subjects (0%), 1 of the 22 POP-I subjects (0%), and 3 of the 16 POP-V subjects (19%). Two of the POP-V subjects (13%) and 1 of the control subjects (5%) possessed foamy macrophages in the tunica intima of a large USL artery (Fig. 4b).

**Table 6:** Occurrence and co-occurrence of CD3, CD68, and CD206 cells in arterial walls. The number of subjects with occurrence and co-occurrence of CD3, CD68, and CD206 cells in the tunica media and adventitia of arteries. The N of each group is indicated. Statistical comparisons for all phenotypes of subjects were performed using R software with required packages indicated where appropriate [20]. Welch’s unequal variances t-test was used when the phenotypes had unequal variances and were of unequal sample size. Analysis of variance with Tukey’s post-hoc pairwise test was used when making multiple comparisons between continuous variables and Pearson's Chi-squared test with Yates' continuity correction for categorical variables (R package ‘RVAideMemoire’). Mean values that significantly differ from one another by group are denoted by letters (a, b, c), *P* ≤ 0.05

Phenotype	*N* per phenotype	*N* of subjects with elevated numbers of CD3 positive cells in or near the arterial wall	*N* of subjects with elevated numbers of CD68 positive cells in or near the arterial wall	*N* of subjects with co-occurrence of CD3 & CD68 positive cells in or near the arterial wall	*N* of subjects with elevated numbers of CD206 positive cells in or near the arterial wall
Control	21	1 (5%) ^a^	1 (5%) ^a^	1 (5%) ^a^	0 (0%) ^a^
POP-A	19	0 (0%) ^a^	0 (0%) ^a^	0 (0%) ^a^	0 (0%) ^a^
POP-I	22	0 (0%) ^a^	1 (5%) ^a^	0 (0%) ^a^	0 (0%) ^a^
POP-V	16	7 (44%) ^b^	9 (56%) ^b^	5 (31%) ^a^	3 (19%) ^a^

Stains for a few of the specialized inflammatory cell types were then undertaken to further define the inflammatory cells which were observed. IHC staining for PD-1 (SupFig 1) was used as a marker for activated lymphocytes [21, 22]. We hypothesized that the T-cells in the arterial walls of arteries with NIH might be activated, however only very low numbers of PD-1 positive cells were observed, and none were detected in the walls of arteries (data not shown).

Macrophages can be broadly divided into two major types with opposing functions; M1 macrophages inhibit cell proliferation and cause tissue damage whereas M2 macrophages promote cell proliferation and tissue repair [23]. IHC staining for CD206 (SupFig 1) was used as a marker for M2 macrophages that were actively promoting wound healing [24]. We hypothesized that the clusters of CD68-positive macrophages and CD3-positive T-cells in the walls of arteries with tunica intima hyperplasia were indicative of previous injuries that were now resolving and that the presence of resolving macrophages (M2) would be evidence in favor of this idea. Indeed, we did observe a few resolving macrophages in the arterial walls of 3 of the 9 POP-V USLs (33%) which contained clusters of CD68 cells, but zero in the arterial walls of control, POP-A, and POP-I USLs (0%) (Table 6).

IHC staining for CD11c (SupFig 1) was used as a marker for dendritic cells, a cell type that activates T-lymphocytes [25]. We hypothesized that there might be dendritic cells in the POP-I tissue due to the broader inflammatory cell presence observed in POP-I USLs, but that there would be very few dendritic cells in the arterial walls of arteries with NIH (POP-V) due to ongoing remodeling. Very few dendritic cells were observed in any of the USLs in this study (Table 5).

IHC staining for CD56, together with a lymphocyte morphology, revealed Natural killer cells (NKT) cells (SupFig1). NKT cells are a subset of restricted T cells now thought to be between the innate and adaptive immune systems [26]. Since NKT cells can have a major regulatory effect upon other immune cell types through their secretion of cytokines, we hypothesized they might amplify or dampen any immune response(s) in the USL. However, very few NKT cells were observed in the USL and therefore images of NKT cells are not shown (Fig. 1). No statistical differences were observed in NKT cell numbers between the control and POP-phenotypes (*p* ≤ 0.05, Table 5).

## Discussion

The immune system is a complex network composed of two systems. The innate immune system is a first line of nonspecific defense consisting of neutrophils, dendritic cells, and natural killer cells that respond within hours to a detrimental process. The adaptive immune response is the secondary line of defense that is mounted over days to weeks and is characterized by macrophages, T cells and B cells. Immune cells are found in circulation and within the extracellular matrix (ECM) of tissues where the ECM composition actively regulates immune processes and signaling. Inflammation has previously been linked to prolapse by our POP-HQ system [16] and by other studies involving both transcript analysis and histology [8–15].

The relative abundance of inflammatory cells in the various prolapse phenotypes identified by our POP-HQ system had demonstrated that the POP-I phenotype, alone, had increased levels of neutrophils [16]. Here, we looked for the broader presence of inflammation in the USL tissue of prolapsing and non-prolapsing subjects. Importantly, the inflammatory cells both in our previous work and here using immunohistological markers for multiple inflammatory cell types were seen infiltrating into the tissue, and not just within vessels in the tissue. Furthermore, in our previous description of the prolapse subgroups, we also looked at the round ligament for a subset of the subjects assessed. We found that the inflammatory signature occurred not only within the USL but also within the matched round ligament specimens [16]. This suggests that the subjects whose USLs exhibit the POP-I phenotype may have a more generalized inflammatory state in tissues within and adjacent to the pelvic floor, although we have no data regarding systemic inflammation. Future examination of systemic inflammatory markers in women that present with advanced POP-Q scores might be useful.

The POP-HQ defined POP-I phenotype separated from the control and other USL phenotypes following PCA on the inflammatory cell quantification data and emphasized the relative difference in inflammatory cell numbers between the POP-I and non-POP-I phenotypes. A closer look at the actual numbers of inflammatory cells in the non-POP-I subjects indicates that inflammation may not necessarily play any role in some of the women with pelvic prolapse. Individuals with the POP-A and POP-V phenotypes of prolapse did not have a statistically significant increase in USL neutrophils, T-lymphocytes, macrophages, or mast cells compared to control USL tissue and do not demonstrate inflammation.

Neutrophils are innate inflammatory cells and are the first inflammatory cell type to respond to detrimental processes. Chronic inflammation follows and the neutrophils are joined by other immune cells that can include macrophages, lymphocytes, and mast cells (and others) [27]. In this study, we have quantified each of these cell types. All the specimens contained neutrophils as indicated by their robust myeloperoxidase positive signal and in addition contain varying quantities of the other inflammatory cell types although this is more heterogeneous. We emphasize that the POP-I phenotype represents only approximately one-third of all USLs evaluated, and that the relationship between inflammatory status and POP development remains to be determined. Li et al. [11] in their single cell transcriptomic analysis of tissue from prolapsed women did not indicate the presence of neutrophils in any of their samples, however their study was of vaginal wall, not USL.

POP-A USL tissue contains a decreased quantity of smooth muscle fibers in the fascicles (Table 2) possibly due to an increased quantity of smooth muscle fiber loss and replacement by collagen [6] and ECM. Macrophages play an important role in remodeling of extracellular matrix through secretion of proteases and degradation of the matrix [28]. Mast cells are also commonly found in tissues undergoing ECM remodeling [29]. However, the POP-A USLs do not contain a statistically significant increased number of either macrophages or mast cells compared to control USLs. This relative absence of cells that would promote degradation of extracellular matrix may suggest a simple increase in the activity of myofibroblast cells as the source of the added POP-A collagen.

The POP-V phenotype has NIH [16]. Aging and injury are the two phenomena thought to be responsible for NIH [30]. Vaginal delivery childbirth is a risk factor for POP, so too are other vaginal traumas, strenuous activity and heavy work lifting [31, 32] however the mechanism by which this occurs is unknown. One possible effect of these activities would be direct trauma / injury to the local vasculature. If injury was responsible for the NIH that we observe in the POP-V USLs, we would expect markers of chronic inflammation such as T-lymphocytes and resolving macrophages to be present focally in the arterial walls as part of the resolution process following that initial injury. Indeed, in 5 of the 16 POP-V USLs (31%) we did observe T-lymphocytes and CD68 positive macrophages in the arterial walls suggesting a previous tissue injury whereas the control and other POP-phenotype USLs had none. However, of the 5 POP-V USLs with increased T-lymphocytes and CD68-positive macrophages, only 3 had CD206-positive (M2) resolving macrophages present. We are unsure why CD206 cells were not present in all 5 of these subjects. Going forward, careful medical histories will be important to further consideration of this hypothesis.

Foamy macrophages were observed in a few of the subjects with NIH. NIH of the artery can be due to either physical trauma or to the trauma of a chronic high fat and high cholesterol diet insult [30]. These few subjects were showing a progression towards atherosclerosis. Again, both past and future medical histories will be important to understand this unexpected observation.

The alternative to injury-driven NIH is aging-driven NIH and the POP-V phenotype are the oldest phenotype; they are statistically older than the control phenotype and trend towards being older than the POP-A and POP-I phenotypes. Therefore, it was important to note that the POP-V phenotype shows no sign of a generalized, age-related increase in inflammatory cells as would be expected with inflammageing [33]. In fact, the POP-V USL tissue, aside from the arteries, is like that of control subjects including similar levels of inflammatory cells, fibrillar collagen, high levels of non-vascular smooth muscle, and low levels of smooth muscle fiber dropout and adipose [16]. The only pathology observed in the POP-V USLs is the presence of NIH.

Dendritic cells play an essential role in the adaptive immune system as professional antigen presenters while also making sure not to react to self or to harmless environmental antigens [34]. Very few dendritic cells were observed in the USLs of our study and most of them were found in the POP-V phenotype. We interpret the relative absence of dendritic cells in the USL tissue of control, POP-A and POP-I subjects as evidence that the serum and USL tissue in these individuals does not contain live organisms or environmental antigens to stimulate the adaptive immune response nor does it contain alterations in the local innate immune system.

Since neutrophils, an innate immune cell type, appear to be of major importance in at least one POP-phenotype (POP-I), however dendritic cells, another innate immune cell type, were virtually absent from USL tissue, we questioned whether the other major innate immune cell type, NKT cells, might play a role in prolapse. NKT cells can have major regulatory effects upon other immune cell types and are able to amplify or dampen immune responses. However, very few NKT cells were found and many USLs did not contain any at all.

How will the data from this study aid future clinicians? Very detailed medical histories have also been taken from all subjects and these are being analyzed with the hope that one or a combination of them will correlate with the relative state of inflammation or the presence of POP. However, we do not yet have an answer.

## Strengths and Weaknesses

The major strengths of our study included utilization of well-defined prolapse phenotypes through application of the semi-quantifiable POP-HQ scoring system followed by the direct immunohistochemical identification and localization of neutrophils, T-lymphocytes, B-lymphocytes, macrophages, and mast cells in the USL-tissues of POP and control subjects, with near 20 subjects per phenotype. Together with previous data, these results reinforce the separation of POP into distinct phenotypes which may lead to actionable strategies based upon the various possible degenerative processes. We further hypothesize that women with different POP-phenotype signatures may have differential risks for complications during treatment of prolapse as well as different incidences of recurrence. Thus, knowledge from studies like this help elucidate the need for a personalized medicine approach to POP. In addition, through our immunohistochemical approach, we have identified which inflammatory cells exist in the vasculature (and are thus excluded from analysis, here) as opposed to simply homogenizing the tissue which would give spurious results.

We acknowledge a few weaknesses with our study. Our study tissue was collected during hysterectomy, and all subjects were experiencing symptomatology leading to their surgery. This means that the control subject group is not representative of the general population. Healthy women seldom have their uterus-cervix block removed. We had access to limited numbers of specimens from older control subjects. This resulted in our inclusion of more postmenopausal specimens from patients with POP than controls. However, we did note that the POP-A and POP-V groups are of similar age, BMI, and parity to the POP-I group and may be better controls for it than the true control group since they have POP whereas the true control group does not. If we use the POP-A or POP-V groups for comparison, the POP-I group still has statistically larger numbers of neutrophils, T-lymphocytes, and macrophages (Table 5). More specimens will be needed to determine how menopausal status influences immune cell trafficking or behavior within the USL. We were also limited in the number of POP-V phenotype specimens available from younger patients. Importantly, we understand that while we have stained for and quantified inflammatory cell types, the presence or absence of an inflammatory cell does not necessarily indicate a functional consequence. Furthermore, we did not measure the levels of serum lymphokines and cytokines and these additional data will be collected in future studies. At present we do not have any means to relate the POP-HQ histological assessment with what is observed when the patient first walks into the office seeking care. Identification of markers able to do this will allow prospective treatment. Lastly, the USL only provides one of the three levels of support for the pelvic organs and its failure is not expected to be solely responsible for the development of all POP. Undoubtedly, failure of other levels of support also contributed to prolapse in patients whose tissue was studied here.

### Summary

In summary, our study is unique in that it makes use of the recently described POP-HQ system to first segregate POP USL tissue into phenotypes and then characterize the presence of inflammatory cells by immunohistochemistry. By doing so we have demonstrated the presence of multiple inflammatory cell types in the POP-I phenotype, a lack of inflammatory cells in the other POP-HQ phenotypes, and we have verified that the inflammatory cells are in the USL tissue as opposed to those within the blood vessels within the USLs. Prior studies that treat the USL as a uniform entity and that homogenize it, before assaying for inflammatory cell proteins or transcripts dismiss the complexity of the tissue and may give inaccurate results. None-the-less, we believe that our study should be replicated on cohorts far larger in size to insure their validity.

## Conclusion

In conclusion, we have directly assessed the presence and localization of inflammatory cells in USL tissue from patients with various subtypes of POP through use of the POP-HQ system and subsequent immunohistochemistry. USL tissue from POP-I phenotype individuals, but not from control, POP-A, or POP-V individuals, demonstrated a statistically significant increase in content of neutrophils, T-lymphocytes, and macrophages (*p* < 0.05). We hypothesize that inflammatory cells may play an important role in the pathology of some but not all subjects with POP.

## Supplementary Information

Below is the link to the electronic supplementary material.

Supplementary file1 (DOCX 5774 KB)

## Data Availability

Complete data used to prepare this manuscript submission have been uploaded to Figshare. No patient identifying information is included. Demographic and histological quantitative and categorical information are provided in an Excel database file link: https://figshare.com/s/365b699d5d8078c90ff0.
