# Supplementary material for: Inflammatory Cells in Control and Prolapsed Uterosacral Ligament Tissue
Source: Reprod Sci. 2024 Jun 21;31(10):3026–38. doi: 10.1007/s43032-024-01618-4 (PMC11438740; doi:10.1007/s43032-024-01618-4)

**Supplemental Figure 1**

Positive (top image of each pair) and negative (bottom image of each pair except Toluidine blue and CD56) control staining for the various markers used in this manuscript. Myeloperoxidase and CD206 staining of colon mucosa; CD3, CD68, CD20, PD-1, and CD11c staining of tonsil germinal centers; CD56 of splenic red pulp, and Toluidine blue staining of mouse epidermis all show the expected staining patterns based upon previous work. Negative controls of toluidine blue and CD56 (not shown) revealed no specific staining.


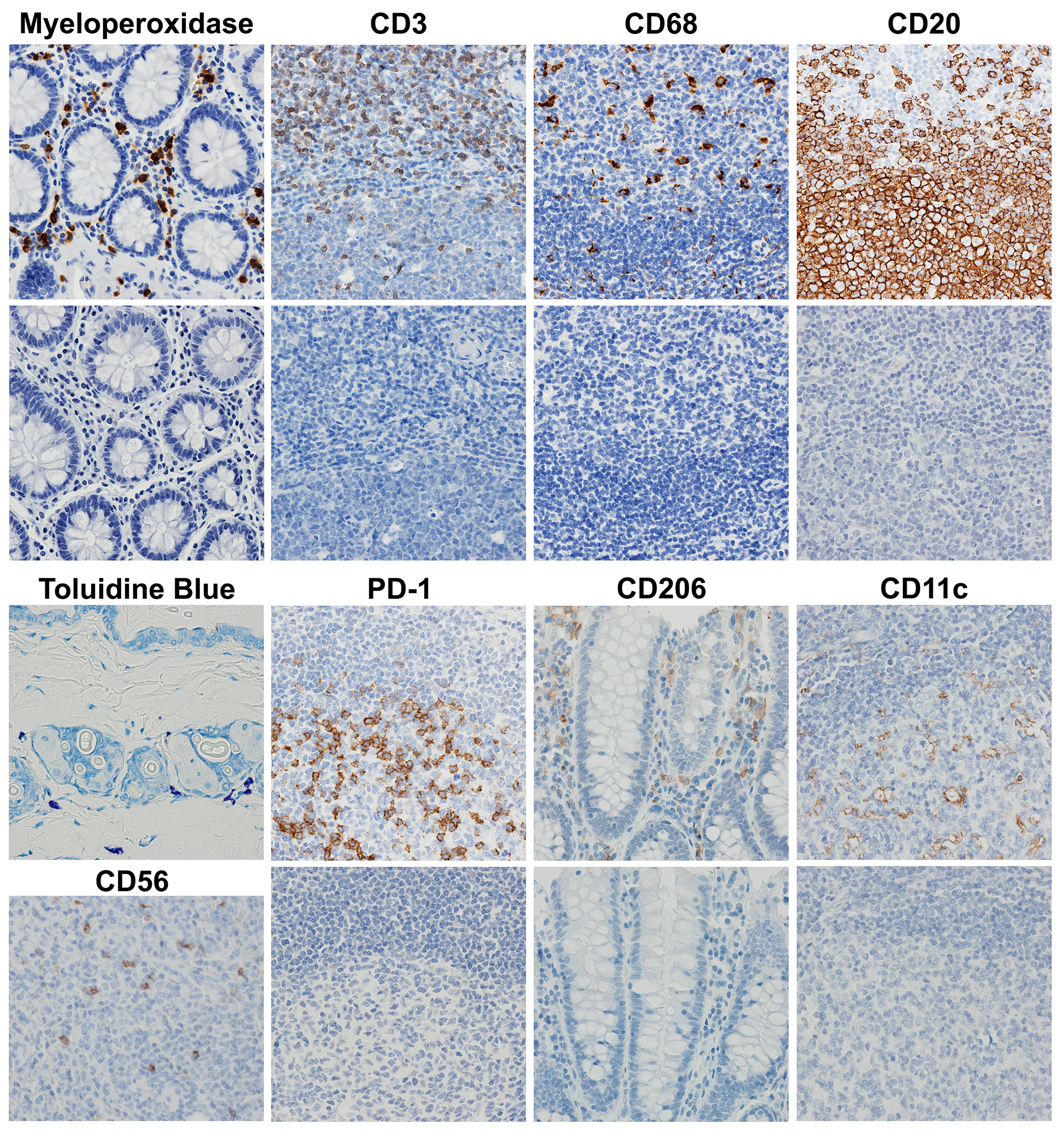

Supplement: Supplementary file 1 — Supplementary file1 (DOCX 5774 KB) [file 43032_2024_1618_MOESM1_ESM.docx]
